# Effects of MyBFF@school, a multifaceted obesity intervention program, on anthropometry and body composition of overweight and obese primary schoolchildren

**DOI:** 10.1186/s12889-024-20724-1

**Published:** 2025-01-14

**Authors:** Ruziana Mona Wan Mohd Zin, Abdul Halim Mokhtar, Abqariyah Yahya, Fuziah Md. Zain, Rusidah Selamat, Zahari Ishak, Muhammad Yazid Jalaludin

**Affiliations:** 1 Department of Pediatrics, Faculty of Medicine, Universiti Malaya, Wilayah Persekutuan Kuala Lumpur, 50603 Kuala Lumpur, Malaysia; 2 Endocrine and Metabolism Unit, Metabolic & Cardiovascular Research Centre, Institute for Medical Research, National Institute of Health (NIH), Ministry of Health, Setia Alam, 40170 NutritionShah Alam, Selangor, Malaysia; 3 Department of Sports Medicine, Faculty of Medicine, Universiti Malaya, Wilayah Persekutuan Kuala Lumpur, 50603 Kuala Lumpur, Malaysia; 4 Faculty of Sports and Exercise Science, Universiti Malaya, Kuala Lumpur, Malaysia; 5 Department of Social and Preventive Medicine, Faculty of Medicine, University of Malaya, Wilayah Persekutuan Kuala Lumpur, 50603 Kuala Lumpur, Malaysia; 6 Department of Pediatrics, Ministry of Health, Hospital Putrajaya, Jalan P9, Pusat Pentadbiran Kerajaan Persekutuan Presint 7Wilayah Persekutuan Putrajaya, 62250 Putrajaya, Malaysia; 7 Nutrition Divison, Federal Government Administrative Centre, Ministry of Health Malaysia, Level 1, Block E3, Complex EWilayah Persekutuan Putrajaya, 62590 Putrajaya, Malaysia; 8 FOSSLA, UCSI University, 56000 Kuala Lumpur, Malaysia

**Keywords:** Childhood obesity, BMI $z$-score, Waist circumference, Percentage body fat, Skeletal muscle mass

## Abstract

**Background:**

Recently, there has been an increase in the prevalence of childhood obesity in Malaysia, raising concerns about increased cardiometabolic morbidity. MyBFF@school is a multifaceted program comprising physical activity, nutritional education, and psychological empowerment introduced to combat childhood obesity in Malaysia. The efficacy of a six-month intervention on the body composition of overweight and obese primary schoolchildren was evaluated.

**Methods:**

This is a school-based, cluster randomized controlled trial involving selected primary schools in Kuala Lumpur, Selangor, and Negeri Sembilan. A total of 1,397 primary-school students aged 9–11 with a body mass index (BMI)$z$-score (corrected for age) greater than + 1 standard deviation based on the World Health Organization 2007 Growth Reference were assigned to intervention ($n=647$) and control ($n=750$) groups. BMI *z*-score, waist circumference (WC), percentage body fat (PBF), and skeletal muscle mass (SMM) were assessed at baseline and after three and six months of the study. Analyses of all outcomes except for the baseline characteristics were conducted according to the intention-to-treat principle.

**Results:**

After three months, there was no significant difference in the BMI *z*-score or PBF between the control and intervention groups, but SMM and WC were significantly higher in the intervention group versus the control group with mean difference of 0.15 kg; 95% confidence interval [CI]: 0.07–0.22, *p* < 0.001 and mean difference of 1.53 cm; 95% confidence interval [CI]: 1.21- 1.85, *p* < 0.001 for SMM and WC respectively. After six months, the intervention group demonstrated a significantly greater reduction in PBF compared to the controls (% mean difference: 0.43%, 95% CI: − 0.73 to − 0.12, *p* < 0.001) as well as a greater increase in SMM (mean difference: 0.28 kg, 95% CI: 0.18–0.37, *p* < 0.001). There was no difference in the BMI *z*-score or WC between the intervention and control groups at six months.

**Conclusions:**

The multicomponent MyBFF@school intervention significantly improved body composition among obese primary schoolchildren in terms of percentage body fat and skeletal muscle mass compared to the control after six months. However, BMI *z*-score and waist circumference measures did not reflect the benefits of this program.

**Trial registration:**

Clinical trial number: NCT04155255, November 7, 2019 (Retrospective registered). National Medical Research Register: NMRR-13–439-16,563. Registered July 23, 2013. The intervention program was approved by the Medical Research and Ethics Committee (MREC), Ministry of Health Malaysia and the Educational Planning and Research Division (EPRD), Ministry of Education Malaysia. It was funded by the Ministry of Health Malaysia.

## Background

Obesity has become a serious global public health threat, placing potentially unsustainable burdens on healthcare systems and leading many countries to declare obesity as a severe chronic disease [1, 2]. In Malaysia, national surveys indicate that childhood obesity under the age of 17 has increased more than doubled in recent years, from 5% in 2006 to 11.8% in 2015, and 14.8% in 2019 [3–6]. Childhood obesity substantially increases the risk of cardiometabolic disorders (e.g., diabetes, hypertension, and heart diseases) in adult life, resulting in an increased risk for premature mortality [7, 8]. In addition, obesity negatively influences children’s quality of life by reducing their physical and psychosocial well-being [9]. The 2017 national survey reported that Malaysian adolescents had a low prevalence of daily fruit and vegetable consumption (23.5%), low physical activity (19.8% physically active), and increased sedentary behavior (50.1% sitting time) [10]. Although the survey does not include younger Malaysian children, the findings may give insight into the status of children overall.

Treating childhood obesity is often time-consuming, complex, and expensive, and current programs often fail to achieve the desired weight-loss goals and health outcomes [11]. Numerous clinical guidelines recommend healthy eating, increased physical activity, and a supportive environment as a primary approach to curb pediatric obesity [12, 13]. Schools are structured environments with facilities and multiple levels of support which is why many weight reduction programs are school based. Further, several systematic reviews suggest that school-based interventions may be the most feasible and effective method for reducing excess weight among larger numbers of schoolchildren [14–16].

Body mass index (BMI) is highly correlated with percentage body fat (PBF) and lean body mass and is, hence, widely used to assess adiposity [17, 18]. However, the validity of BMI as a measure of adiposity in children is limited as any increase in the BMI throughout childhood is primarily due to a rise in lean body mass and stature rather than fat mass [19]. Therefore, multiple outcome metrics are required to comprehensively assess health program outcomes. This study aims to evaluate the benefits of the school-based lifestyle intervention program “My Body is Fit and Fabulous at School” (MyBFF@school) on BMI, body composition, and other metrics of overweight and obese primary schoolchildren.

## Methods

### Study design and population

**Fig. 1:**
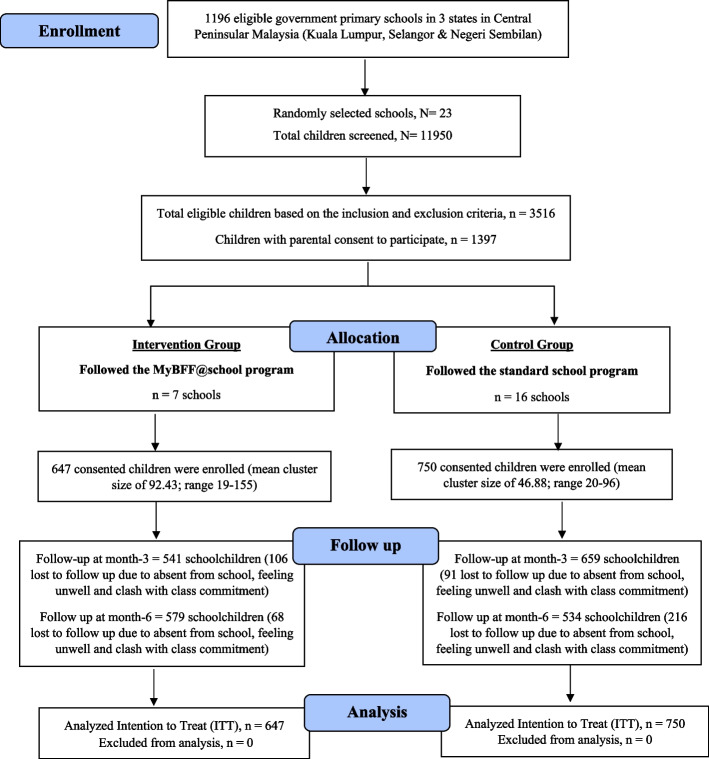
CONSORT diagram for central component in MyBFF@school

This was a school-based, cluster randomized controlled trial (Fig. 1) evaluating the effect of MyBFF@school on body composition among overweight and obese primary schoolchildren after three and six months. MyBFF@school is a multifaceted obesity intervention program that incorporates physical activity, healthy eating promotion, and psychological empowerment. All public primary schools in the central region of Peninsular Malaysia (Selangor, Kuala Lumpur and Negeri Sembilan) were eligible to participate in this study. The eligible schools were randomly selected using proportionate random sampling to ensure sufficient representation of multi-ethnic populations in the study sample and were stratified according to school type (national or vernacular schools) and their location (urban or rural schools). Then the selected schools were randomly assigned to either control schools or intervention schools.A total of 23 primary schools in Kuala Lumpur, Selangor, and Negeri Sembilan were randomized into intervention and control schools. Inclusion criteria for the study were schoolchildren aged 9 to 11 years old with a BMI for age of more than + 1 SD based on the WHO 2007 Growth Reference (overweight and obese). The exclusion criteria were BMI for age below and/or equal to + 1 SD, with physical or mental disability, medical conditions that prevented their participation in moderate to vigorous physical activities, co-morbidities that may interfere with the study (such as diagnosed type 2 diabetes mellitus, hypertension, nephritic syndrome, epilepsy, congenital heart disease and skeletal anomalies), or a requirement for steroids, anti-epileptic treatments, or methylphenidate. The selected intervention schools delivered the MyBFF@school program from mid-February to mid-August 2016, whereas the control schools followed the standard national school curriculum. The method is described in detail in another article (Mokhtar AH, Wan Mohd Zin RM, Yahya A, Md. Zain F, Selamat R, Ishak Z, Jalaludin MY: Rationale, design and methodology of My Body is Fit and Fabulous at school (MyBFF@school) study: a multi-pronged intervention program to combat obesity among Malaysian schoolchildren, unpublished). The sample size estimation was established based on the main outcome parameter of the mean difference in percentage body fat. The investigators first computed a sample size for a standard randomized controlled trial (RCT) (individuals’ randomization) (N) where independence of samples was assumed. In this standard RCT, to achieve 80% power at a 5% significance level, a minimal number required to detect a mean difference of 0.35 in percentage body fat was 804 (402 per arm). This was based on the investigators’ unpublished findings on changes of percentage body fat and attrition rate in a pilot study [20]. A total of 1,397 primary schoolchildren assented and consented by a parent/guardian participated in the study. Body composition was measured at baseline and after three and six months of the intervention (or control conditions).

### Ethics statement

This study was approved by the Medical Research and Ethics Committee (MREC), Ministry of Health Malaysia (NMRR-13–439-16,563). Written informed consent and assent were obtained from parents/guardians and children, respectively, prior to the start of the program. All tests and procedures were conducted according to guidelines approved by the MREC.

### Anthropometric and body composition assessment

Anthropometric measurements were performed by trained personnel, and health examinations were performed by medical officers and pediatricians. Standing height was measured without shoes to the nearest 0.1 cm using a calibrated stadiometer (Seca 217; Seca, Hamburg, Germany). Body weight and body composition were measured in light clothing without shoes and socks to the nearest 0.1 kg using a pre-calibrated body impedance analyzer (InBody 720; InBody, Seoul, Korea). PBF was measured to the nearest 0.1% and SMM to the nearest 0.1 kg using the same body impedance analyzer. Noradilah et al. have shown that the BIA-based prediction equation from the manufacturer had good agreement with dual-energy X-ray absorptiometry (DXA) and can be used to measure body composition at a population level in Malay children. All BIA equations showed reasonable agreement with DXA. The best agreement was seen for BIA Manufacturers that had a relatively small mean bias and acceptable limits of agreement with no apparent extreme bias compared with other BIA equations [21]. WC was measured twice to the nearest 0.1 cm over the skin midway between the 10th rib and the iliac crest at the end of normal expiration using a non-extensible tape (Seca 201), and the mean was recorded. Pubertal/sexual maturity was assessed following Tanner’s staging scale criteria [22, 23].

### Definition of measures

BMI $z$-score was calculated using the WHO AnthroPlus 2007 software. Overweight, obese, and morbidly obese were defined as BMI *z*-scores greater than 1, 2, and 3 SDs, respectively, for age and gender according to the WHO BMI chart (2008). Tanner’s staging was assessed by showing standardized Tanner staging pictures to each child. Stage 1 external genitalia development and breast development for boys and girls, respectively, were classified as pre-pubertal, whereas stage 2 and above were defined as pubertal. Abdominal obesity was defined as WC measurement ≥ 90th percentile of the Malaysian WC chart [24].

### Statistical analysis

All data analyses were conducted using SPSS Statistics for Windows (Version 24.0; IBM Corp., Armonk, NY, USA). The normality of continuous parameters was evaluated using the Kolmogorov–Smirnov test. Means and SDs were calculated for continuous variables. Means at baseline were compared between the intervention and the control groups using independent samples *t*-tests. Baseline categorical variables were compared between groups using the chi-squared test. Within-group and between-group differences were analyzed using a one-way analysis of covariance (ANCOVA) with group (intervention versus control) as the independent variable. A $p$-value of < 0.05 (two-tailed) was considered significant for all tests. All analyses except for those of the baseline characteristics were performed according to the intention-to-treat principle using the multiple imputation method.

## Results

A total of 1,397 primary schoolchildren aged 9–11 were enrolled in the MyBFF@school program, with 647 children assigned to the intervention group and 750 children assigned to the control group. The baseline characteristics of the intervention and control groups are summarized in Table 1. The gender ratio, pubertal status, PBF, and SMM did not differ between the groups (all $p>0.05$). The mean age was slightly but significantly higher in the control group compared to the intervention group (9.92 versus 9.81 years). In addition, the mean WC at baseline was slightly but significantly higher in the control group (75.6 cm versus 74.4 cm), but the rate of abdominal obesity as defined by the 90th percentile WC cutoff did not differ between the groups. Similarly, the distribution of overweight, obese, and morbidly obese participants did not differ between the control and the intervention groups at baseline. Ethnic distribution differed slightly between the groups (*p* = 0.04) with the intervention group having a higher percentage of Malay and other ethnicities. Finally, the control group comprised a significantly higher proportion of urban participants. The significant differences in ethnic and location (urban–rural) participants could not be avoided as the randomization was done on the schools through proportionate random sampling, and not the schoolchildren (Mokhtar AH, Wan Mohd Zin RM, Yahya A, Md. Zain F, Selamat R, Ishak Z, Jalaludin MY: Rationale, design and methodology of My Body is Fit and Fabulous at school (MyBFF@school) study: a multi-pronged intervention program to combat obesity among Malaysian schoolchildren, unpublished).

**Table 1:** Baseline characteristics of the control and intervention groups

	**Control**	**Intervention**	${\varvec{p}}$**-value**
	**(** ${\varvec{n}}=750$**)**	**(** ${\varvec{n}}=647$**)**	
**Age, mean (SD)**^**a**^	9.92 (0.82)	9.81 (0.84)	0.01
**Gender (** ${\varvec{n}}$**, %)**^**b**^			
**Male**	403 (53.7)	348 (53.8)	0.98
**Female**	347 (46.3)	299 (46.2)	
**Ethnicity (** ${\varvec{n}}$**, %)**^**b**^			
**Malay**	376 (68.7)	445 (70.6)	0.04
**Chinese**	108 (19.7)	106 (16.8)	
**Indian**	50 (9.1)	47 (7.5)	
**Other**	13 (2.4)	32 (5.1)	
**Region (** ${\varvec{n}}$**, %)**^**b**^			
**Urban**	508 (67.7)	357 (55.2)	< 0.001
**Rural**	242 (32.3)	290 (44.8)	
**Pubertal status (** ${\varvec{n}}$**, %)**^**b**^			
**Prepubertal**	494 (66.1)	429 (66.6)	0.85
**Pubertal (Tanner stage ≥ 2)**	253 (33.9)	215 (33.4)	
**BMI ** ${\varvec{z}}$**-score, mean (SD)**^**a**^	2.30 (0.82)	2.27 (0.80)	0.51
**WC (cm), mean (SD)**^**a**^	75.66 (9.60)	74.40 (9.60)	0.02
**PBF (%), mean (SD)**^**a**^	38.09 (6.54)	37.50 (6.59)	0.08
**SMM (kg), mean (SD)**^**a**^	14.73 (2.92)	14.59 (2.91)	0.28
**BMI ** ${\varvec{z}}$**-score status (** ${\varvec{n}}$**, %)**^**b**^			
**Overweight > + 1SD**	288 (38.4)	265 (41.0)	0.62
**Obese > + 2SD**	327 (43.6)	270 (41.7)	
**Morbidly obese > + 3SD**	135 (18.0)	112 (17.3)	
**Abdominal obesity (** ${\varvec{n}}$**, %)**^**b**^			
**WC < 90th centile**	242 (32.5)	217 (33.9)	0.60
**WC > 90th centile**	502 (67.5)	424 (66.1)	

*SD* standard deviation, *BMI* body mass index, *WC* waist circumference, *PBF* percentage body fat, *SMM* skeletal muscle mass

^a^Means at baseline were compared using independent samples *t*-tests (intervention versus control groups)

^b^Baseline categorical variables were compared between groups using the chi-squared test

Table 2 summarizes the mean within-group changes and between-group differences in anthropomorphic measures and body composition after three and six months. After three months, the control group demonstrated a significant reduction in the BMI $z$-score ($p=0.01$), lower PBF ($p<0.001$), and higher SMM ($p<0.001$) compared to the baseline after adjusting for gender. In contrast, the intervention group showed no change in the BMI $z$-score but a significantly larger mean WC ($p<0.001$) compared to the baseline. However, both groups demonstrated similar reductions in PBF and increase in SMM (all $p<0.001$). Between-group comparisons after three months revealed significantly larger changes in the WC and SMM among the intervention group compared to the control group (both $p<0.001$) after adjusting for gender. Nevertheless, there was no difference in PBF changes between groups.

**Table 2:** Within-group and between-group changes in body composition metrics during the intervention period

Body composition	Visit	Group	Change within-group (visit, baseline)	$p$-value^a^	Change between group (intervention, control)	$p$-value^*a*^
			**Mean (95% CI)**		**Mean (95% CI)**	
**BMI ** $z$**-score**	**Month 3**	Control	− 0.02 (− 0.03, 0.00)	0.01	+ 0.01 (− 0.01, 0.03)	0.49
		Intervention	− 0.01 (− 0.03, 0.01)	0.58		
	**Month 6**	Control	+ 0.01 (− 0.01, 0.03)	1.0	− 0.01 (− 0.04, 0.02)	0.37
		Intervention	0.00 (− 0.03, 0.02)	1.0		
**WC (cm)**	**Month 3**	Control	+ 0.03 (− 0.23, 0.30)	1.0	+ 1.53 (1.21, 1.85)	< 0.001
		Intervention	+ 1.63 (1.34, 1.92)	< 0.001		
	**Month 6**	Control	+ 1.99 (1.64, 2.33)	< 0.001	+ 0.33 (− 0.06, 0.71)	0.35
		Intervention	+ 2.42 (2.09, 2.75)	< 0.001		
**SMM (kg)**	**Month 3**	Control	+ 0.63 (0.57, 0.69)	< 0.001	+ 0.15 (0.07, 0.22)	< 0.001
		Intervention	+ 0.77 (0.71, 0.84)	< 0.001		
	**Month 6**	Control	+ 1.36 (1.28, 1.44)	< 0.001	+ 0.28 (0.18, 0.37)	< 0.001
		Intervention	+ 1.63 (1.54, 1.72)	< 0.001		
**PBF (%)**	**Month 3**	Control	− 0.71 (− 0.89, − 0.52)	< 0.001	+ 0.02 (− 0.20, 0.25)	0.42
		Intervention	− 0.65 (− 0.86, − 0.43)	< 0.001		
	**Month 6**	Control	− 0.33 (− 0.59, − 0.08)	0.006	− 0.43 (− 0.73, − 0.12)	0.007
		Intervention	− 0.71 (− 0.99, − 0.42)	< 0.001		

*BMI* body mass index, *CI* confidence interval, *WC* waist circumference, *PBF* percentage body fat, *SMM* skeletal muscle mass

^a^Within-group and between-group differences were analyzed using one-way analysis of covariance (ANCOVA) with group (intervention versus control) as the independent variable

After six months, neither group demonstrated a significant improvement in the BMI $z$-score, whereas both groups demonstrated significant increases in the WC and SMM and significant reductions in the PBF. Between-group comparisons after six months indicated no difference in the BMI $z$-score or mean WC change, but the intervention group demonstrated a greater increase in the SMM and a greater reduction in the PBF compared to the control group ($p<0.001$ and $p=0.007$, respectively).

## Discussion

The MyBFF@school program did not improve the BMI z-score among the intervention group after three or six months. In contrast, the control group had a significant reduction of BMI z-score at month 3, but no further improvement in month 6. Although BMI is the most often used parameter to categorize obesity, the outcomes of weight-loss programs that utilized BMI z-score yielded inconsistent results, even after controlling for various factors. For instance, few studies have reported a significant reduction in the BMI z-score after three, six, and twelve months of the multifaceted intervention program [25–27]. On the contrary, Seo et al. found no significant differences between the intervention and control groups following a similar multiple-component intervention program for children with obesity [28], which is consistent with our findings. Nevertheless, BMI is not a strong predictor of body fat composition or adiposity among overweight and obese children [29, 30]. The BMI z-score has been reported to perform poorly in identifying slight changes in body composition and is weakly correlated with other adiposity parameters such as WC, skinfold thickness, and fat mass, especially among children with obesity [31]. A similar 12-week intervention study for children resulted in significantly reduced body fat mass but no significant change in BMI [32]. Therefore, multiple adiposity-related outcomes must be included when evaluating weight-loss programs for children. In this study, the MyBFF@school program demonstrated significant efficacy in reducing PBF and increasing SMM compared to the controls despite having no effects on abdominal obesity or BMI.

Reduction in PBF is a relatively consistent finding following such multifaceted interventions, with significant reductions reported among overweight and obese children after three months [33], five months [34] and six months [26]. It has been reported that PBF is significantly and positively correlated with insulin resistance [35], a major pathogenic factor in diabetes, and increases the odds of cardiovascular risk factor clustering in children [36]. Moreover, children with high fat mass were found to exhibit a higher risk of bone fractures and to show aberrant skeletal development [37]. PBF was also identified as a predictor of depression and poor health-related quality of life among children aged 8–17 years [38]. Hence, a reduction in PBF even in the absence of a relative BMI decrease could improve the physiological and psychological wellbeing of children.

A significant increase in SMM was observed in both the control and intervention groups at three and six months, consistent with ongoing growth and development, but the increase was greater in the intervention group. SMM plays a major role in the pathogenesis of metabolic syndrome as it constitutes the largest insulin-sensitive tissue in the body [39, 40]. It was found that low muscle mass has been associated with low cardiorespiratory fitness in adults [41], hence, strengthening SMM in children might be beneficial towards cardiovascular health [42]. Among other benefits, increased SMM is positively associated with bone mineral density [43], therefore, high SMM is a protective factor against fractures even among children with high levels of physical activity [44]. In the MyBFF@school study, it is possible that the SMM gain in both intervention and control groups is contributed by puberty. It is known that puberty may affect body composition namely fat free mass and skeletal mass. The boys gain a significant increase in SMM while the girls gain more in fat mass [45, 46]. Future intervention studies should consider the possible confounding effect of puberty on the outcome.

Both groups demonstrated a significant increase in WC, and this increase was greater in the intervention group, in accord with a similar study by Amini et al. [25]; however, others reported significant reductions after six months [26]. As children grow, changes in body composition and increasing stature might contribute to the increase in WC. WC normogram of Malaysian children also shows increasing WC by age, consistent with child growth [24]. This growth might include increases in muscle mass, bone mass and overall body size, all of which may have an impact on the WC measurements. Therefore, instead of solely monitoring changes in WC, it is more beneficial to classify abdominal obesity using the 90th percentile for a more precise evaluation of weight-loss intervention study among children with obesity. While the intervention did not improve a key metric of abdominal obesity in this study, overall adiposity was reduced to a greater extent compared to the control group.

A notable limitation of this study is the short intervention period. Although there is a lack of consensus regarding the minimum duration needed to show an effect for school-based interventions [47], a longer-term follow-up is recommended to assess sustainability. Llargues et al. conducted a cluster randomized controlled trial on 16 primary schools and found that the intervention group exhibited a significantly smaller increase in BMI compared to the control group after six years [48]. Therefore, our intervention may have long-term health benefits not immediately manifested by BMI changes. Second, this study did not include a secondary home setting or parent outreach during the intervention period. Parents may provide a so-called obesogenic environment for their children, so it is critical to extend the lifestyle changes of MyBFF@school to the home setting. Indeed, such interventions were found to be most effective when including both a home and a community setting [49].

Despite these limitations, this program has several important strengths. First, the program is a multicomponent intervention targeting diet, physical activity, and psychological wellbeing, and it was found in a systematic review of school-based studies that such multi-component interventions are the most effective [50]. In addition, this program included a unique psychological component to encourage self-efficacy and empowerment in health-related decisions. The strengths of the study included a relatively large ethnically diverse sample and the inclusion of a morbidly obese population, whereas most previous studies have excluded this population or did not distinguish obesity from morbid obesity.

## Conclusion

The multicomponent MyBFF@school intervention significantly improved body composition among obese primary schoolchildren in terms of percentage body fat and skeletal muscle mass compared to the control after six months. However, BMI *z*-score and waist circumference measures may not reflect the benefits of this program.

## Data Availability

All relevant data are within the paper.

## Abbreviations

ANCOVA: Analysis of covariance
ANOVA: Analysis of variance
BMI: Body mass index
CI: Confidence interval
MREC: Medical Research and Ethics Committee
PBF: Percentage body fat
SD: Standard deviation
SMM: Skeletal muscle mass
WC: Waist circumference
WHO: World Health Organization
